# Comparative effectiveness of genotype-guided and empirical bismuth-containing quadruple therapy for *Helicobacter pylori* eradication: a retrospective study

**DOI:** 10.3389/fphar.2026.1875484

**Published:** 2026-09-03

**Authors:** Pan Wang, Mengyuan Yang, Xiaolin Zhao, Haiou Ding, Jianping Cheng

**Affiliations:** 1 Department of Pharmacy, Civil Aviation General Hospital, Beijing, China; 2 Department of Gastroenterology, Civil Aviation General Hospital, Beijing, China

**Keywords:** bismuth-containing quadruple therapy, clarithromycin, genotype-guided therapy, *Helicobacter pylori*, levofloxacin, potassium-competitive acid blocker

## Abstract

**Objective:**

To compare the efficacy of genotype-guided versus empirical bismuth-containing quadruple therapy (BQT) for *Helicobacter pylori* (*H. pylori*) eradication and to identify factors influencing treatment success.

**Methods:**

This single-center retrospective study included 616 patients with *H. pylori* infection who received 14-day BQT. All regimens comprised bismuth potassium citrate 2.6 g, a proton pump inhibitor (PPI, esomeprazole 20 mg or ilaprazole 5 mg) or a potassium-competitive acid blocker (P-CAB, keverprazan 20 mg or tegoprazan 50 mg), and antibiotics selected from amoxicillin, clarithromycin, levofloxacin, furazolidone, tetracycline, metronidazole, or doxycycline. In the empirical group, antibiotic selection was at the physician’s discretion. In the genotype-guided group, selection was tailored to qPCR-based detection of clarithromycin and levofloxacin resistance. Eradication was confirmed by urea breath test at least 4 weeks after treatment.

**Results:**

Eradication rates were significantly higher with genotype-guided therapy (90.8% vs. 81.7%; OR 2.03, 95% CI 1.23–3.36; *P* = 0.006). In the empirical group, P-CAB-based regimens achieved higher eradication than PPI-based regimens (87.9% vs. 76.3%; OR 2.24, 95% CI 1.38–3.58; *P* < 0.001), whereas the difference was not significant in the genotype-guided group (93.5% vs. 86.9%; OR 2.12, 95% CI 0.94–4.78; *P* = 0.070). Among patients receiving genotype-guided therapy, dual resistance to clarithromycin and levofloxacin was associated with the lowest eradication rate (82.9%), significantly lower than that in the fully susceptible group (94.1%; OR 0.30, 95% CI 0.11–0.86; *P* = 0.025). Current alcohol consumption was an independent risk factor for eradication failure (OR 0.39, 95% CI 0.16–0.92; *P* = 0.032).

**Conclusion:**

Genotype-guided BQT significantly improved *H. pylori* eradication compared with empirical BQT. The superiority of P-CAB over PPI was most evident in the empirical setting. Dual clarithromycin and levofloxacin resistance and alcohol consumption remained key determinants of treatment failure even with genotype-guided therapy. However, the precision of estimates for single-drug resistance subgroups was limited by small sample sizes and should be interpreted cautiously.

## Introduction

*Helicobacter pylori* (*H. pylori*) is a Gram-negative bacterium that colonizes the gastric mucosa of approximately half of the world’s population (Ailloud et al., 2019). Chronic *H. pylori* infection is the primary etiological factor for chronic gastritis, peptic ulcer disease, gastric mucosa-associated lymphoid tissue lymphoma, and gastric adenocarcinoma (Murakami et al., 2016). In recognition of its carcinogenic potential, the World Health Organization classified *H. pylori* as a class I carcinogen in 1994 (Bauer et al., 2020). A growing body of evidence supports the cost-effectiveness of *H. pylori* eradication for gastric cancer prevention, particularly when implemented before the onset of pre-neoplastic changes in high-incidence regions (Wang et al., 2022; Kowada, 2023; Li Y. et al., 2025).

Clarithromycin, a cornerstone of triple therapy, has seen resistance rates rise to alarming levels worldwide. A study conducted in the United States and Europe reported clarithromycin resistance in 22.2% of *H. pylori* strains, while recent meta-analyses have documented resistance rates of 31.5% for clarithromycin and 37.6% for levofloxacin (Ho et al., 2022; Mégraud et al., 2023). According to the fifth Chinese National Consensus Report on the management of *H. pylori* infection (Liu et al., 2018), resistance rates to metronidazole, clarithromycin, and levofloxacin were reported at 60%–70%, 20%–38%, and 30%–38%, respectively, while resistance to amoxicillin, furazolidone, and tetracycline remained comparatively low at 1%–5%. These trends have rendered clarithromycin-based triple therapy increasingly ineffective, with eradication rates falling below the acceptable threshold of 80% in many regions (Abadi and Kusters, 2016).

In response to these challenges, the Maastricht VI/Florence Consensus recommended that in areas with high clarithromycin resistance (>15%), bismuth-containing quadruple therapy (BQT) should be employed as first-line treatment (Malfertheiner et al., 2022). This recommendation was adopted by the fifth Chinese National Consensus Report, which established 14-day BQT as the standard first-line regimen in China (Liu et al., 2018; Ding et al., 2022). This approach combines a proton pump inhibitor (PPI) or potassium-competitive acid blocker (P-CAB) with bismuth and two antibiotics, typically including amoxicillin in combination with clarithromycin, levofloxacin, doxycycline, tetracycline, or furazolidone. The 2022 Chinese National Clinical Practice Guideline further reinforced this recommendation, reporting average eradication rates of 81.3%–83.6% for 14-day BQT (Zhou et al., 2022). In 136 *H. pylori*-infected patients without susceptibility testing, levofloxacin-based regimen achieved a 97.3% eradication rate with favorable tolerability, suggesting potential superiority over clarithromycin-based regimen (82.2%) (Salehi et al., 2025). However, real-world data have revealed that achieving these target eradication rates remains challenging. In a recent retrospective study from our center encompassing 3,340 patients, the overall eradication rate was 68.1%, substantially lower than the rates reported in controlled clinical trials (Cheng et al., 2024).

Despite the widespread adoption of bismuth-containing quadruple therapy, the empirical selection of antibiotics remains problematic in the context of high and geographically variable resistance patterns (Chey et al., 2024). The inability to reliably predict resistance to clarithromycin and levofloxacin without susceptibility testing frequently results in the use of regimens containing antibiotics to which the infecting strain is resistant, contributing to treatment failure (Zivarifar and Keikha, 2025). PCR-based assays targeting 23S rRNA mutations for clarithromycin resistance and gyrA mutations for levofloxacin resistance can offer rapid, accurate, and clinically implementable results (Šamanić et al., 2023; Fang et al., 2024). These assays have also been validated using non-invasive fecal samples, broadening their clinical applicability (Peng et al., 2025). Clinical studies have demonstrated that genotype-guided tailored therapy achieves satisfactory eradication rates, and in pooled analyses of first-line treatment, it is superior to empirical triple therapy while comparable to empirical BQT (Li et al., 2023).

Despite the growing body of evidence supporting genotype-guided therapy, the interaction between resistance patterns and acid suppressant selection within the context of genotype-guided therapy remains unclear. To address these gaps, we conducted a single-center retrospective study comparing genotype-guided versus empirical BQT for *H. pylori* eradication. The present study aimed to compare eradication rates between genotype-guided therapy and empirical BQT, evaluate the influence of acid suppressant type on eradication outcomes within each treatment strategy, characterize the impact of distinct antimicrobial resistance patterns on treatment success in the genotype-guided group, and identify independent predictors of eradication failure. Collectively, these findings offer clinically actionable insights for optimizing *H. pylori* eradication in the context of increasing antimicrobial resistance.

## Methods

### Study design and ethical approval

This single-center retrospective cohort study adhered to the applicable 2007 Strengthening the Reporting of Observational Studies in Epidemiology (STROBE) guidelines (Vandenbroucke et al., 2007). The study protocol was approved by the Institutional Ethics Board of Civil Aviation General Hospital, Beijing, China (Approval No. KY2026-026-01). Because patient information was collected anonymously and no interventions were performed, the Institutional Ethics Board waived the requirement for individual informed consent.

### Study population and data collection

Detailed patient information was extracted from the hospital’s electronic medical record system, including endoscopic reports, prescription records, and laboratory results. Data collected included age, sex, lifestyle characteristics, treatment regimens, and post-treatment UBT results. Smoking was defined as current daily or weekly consumption of ≥1 cigarette per day for ≥6 months; alcohol consumption was defined as intake of ≥50 g of pure alcohol per week for ≥6 months (Manzoli et al., 2013; Wang et al., 2022). All patient data were anonymized and assigned unique identifiers to ensure privacy protection. Two independent clinicians verified the treatment regimens and outcome data to ensure accuracy and consistency. All patients were enrolled consecutively during the study period to minimize temporal bias. The decision to perform genotype-guided testing and tailor therapy accordingly was made at the discretion of the attending physician, based on clinical judgment, patient preference, and the availability of the qPCR testing service at our center. In the empirical group, antibiotic selection was based on the physician’s clinical experience, local resistance patterns, and consideration of patient-specific factors such as allergy history and prior antibiotic exposure.

Inclusion criteria were: (1) availability of demographic, clinical, and prescription data; (2) confirmed diagnosis of *H. pylori* infection by positive UBT; (3) receipt of 14-day BQT comprising a PPI or P-CAB, bismuth, and two antibiotics, with antibiotic combinations and dosages conforming to the Fifth Chinese National Consensus Report on the management of *H. pylori* infection; for the genotype-guided group, regimen selection was additionally informed by genotypic resistance testing results; and (4) post-treatment confirmation of *H. pylori* eradication status by UBT performed 4 weeks after therapy completion. Exclusion criteria were: (1) loss to follow-up or non-adherence to the prescribed regimen; (2) prior history of *H. pylori* eradication therapy; (3) negative *H. pylori* detection by qPCR in gastric mucosal samples; and (4) receipt of additional medications during the treatment period.

### *Helicobacter pylori* genotypic resistance detection

During gastroscopy, two gastric biopsy specimens were obtained from the gastric antrum and corpus of each patient using disposable biopsy forceps. All specimens were processed following standardized stabilization procedures. Samples were snap-frozen in liquid nitrogen within 30 min of collection and subsequently stored at −80 °C. All samples were transported to the laboratory within 72 h of collection.

Genomic DNA was extracted from gastric mucosal samples using the QIAamp DNA Mini Kit (56304, Qiagen, Hilden, Germany) following the manufacturer’s instructions. A commercial qPCR detection kit (Jiangsu Mole Bioscience Co., Ltd., Taizhou, Jiangsu, China) was employed to detect antibiotic resistance-associated gene mutations. Clarithromycin resistance was determined by detecting point mutations in the 23S rRNA gene at positions A2143C, A2143G, and A2144G. Levofloxacin resistance was determined by detecting mutations in the gyrA gene at positions C260T, A261G, A261A, C271A, C271T, and A272G. Each run included positive and negative controls as specified by the manufacturer. Resistance was defined by the presence of any of the target mutations, with interpretation based on the amplification curves and cycle threshold (Ct) values according to the kit’s instructions. This qPCR assay has been previously validated in our center (Cheng et al., 2025).

### Treatment regimens

All patients received 14-day BQT. In the empirical therapy group, the antibiotic combination was selected at the discretion of the attending physician based on clinical experience and routine practice. In the genotype-guided therapy group, antibiotic selection was tailored to the qPCR-based resistance results. Patients with resistance to either clarithromycin or levofloxacin received BQT avoiding the corresponding resistant antibiotic. For patients with dual resistance (resistance to both clarithromycin and levofloxacin), recommended alternatives included amoxicillin-furazolidone, tetracycline-metronidazole, or doxycycline-metronidazole combinations, based on individual tolerance and absence of contraindications. Patients with susceptibility to both antibiotics had no regimen restrictions.

All BQT regimens comprised bismuth potassium citrate granules 2.6 g three times daily, an acid suppressant administered twice daily, and two antibiotics. Acid suppressants included standard-dose proton pump inhibitors (esomeprazole 20 mg or ilaprazole 5 mg) or potassium-competitive acid blockers (keverprazan 20 mg or tegoprazan 50 mg). Antibiotics were selected from the following agents: amoxicillin 1,000 mg twice daily, clarithromycin 500 mg twice daily, levofloxacin 500 mg once daily, furazolidone 100 mg twice daily, tetracycline 500 mg three or four times daily, metronidazole 400 mg three or four times daily, and doxycycline 100 mg twice daily.

### Study outcomes

Eradication rates were compared between the genotype-guided and empirical therapy groups. Within each group, eradication rates were further stratified by acid suppressant type (P-CAB versus PPI). In the genotype-guided group, eradication rates were evaluated according to resistance patterns, categorized as fully susceptible (susceptible to both clarithromycin and levofloxacin), clarithromycin-resistant only, levofloxacin-resistant only, and dual-resistant (resistant to both antibiotics). Factors associated with treatment failure, including demographic characteristics, lifestyle factors, and treatment-related variables, were also assessed.

### Statistical analysis

Based on prior literature reporting eradication rates of approximately 85%–90% for genotype-guided therapy and 75%–80% for empirical BQT (Zhou et al., 2022; Li et al., 2023). A sample size calculation was performed using a two-sided chi-square test with a significance level (α) of 0.05 and statistical power (1-β) of 80%. This calculation indicated that a minimum of 152 patients per group would be required to detect the specified difference. To account for potential exclusions and loss to follow-up, we aimed to enroll at least 350 patients in the empirical group and 250 patients in the genotype-guided group, which were ultimately achieved (n = 355 and n = 261, respectively). Eradication rates were calculated on a per-protocol analysis, including only patients who completed the 14-day treatment regimen and had post-treatment UBT results available. Patients lost to follow-up or with documented non-adherence were excluded per the study criteria. Statistical analyses were performed using SPSS version 26.0 (SPSS Inc., Chicago, IL, United States). Continuous variables were expressed as mean ± standard deviation (SD). Categorical variables were presented as frequencies and percentages. Univariate logistic regression analysis was initially conducted to identify potential predictors of eradication failure. Variables in univariate analysis, along with clinically relevant variables (age, sex, smoking, alcohol consumption, acid suppressant type, and resistance pattern), were entered into multivariate logistic regression models using the enter method to identify independent predictors of treatment failure. Results were reported as odds ratios (OR) with 95% confidence intervals (CI). A two-sided *P*-value < 0.05 defined statistical significance. The full covariate set included in all multivariate models comprised age, sex, smoking status, alcohol consumption, acid suppressant type, and treatment strategy when assessing overall treatment effect. In subgroup analyses restricted to the genotype-guided group, the covariate set additionally included resistance pattern. Variables were selected for inclusion based on a two-stage approach: first, all variables with a univariate *P* < 0.10 were considered for entry; second, clinically relevant variables were forced into the models regardless of their univariate significance to control for potential confounding. There were no missing data for any of the key covariates and all patients met the inclusion criteria for complete data availability. Interaction terms between treatment strategy and acid suppressant type, as well as between resistance pattern and acid suppressant type within the genotype-guided group, were assessed by including product terms in the logistic regression models. To quantify the potential impact of unmeasured confounding on our primary findings, we calculated the E-value (VanderWeele and Ding, 2017) for the observed OR of genotype-guided versus empirical therapy. E-values were calculated for both the point estimate and the lower bound of the 95% confidence interval to assess robustness against hidden bias.

## Results

### Patient baseline characteristics

This study included 616 patients with documented *H*. *pylori* infection, including of 355 patients in the empirical therapy group and 261 patients in the genotype-guided therapy group. No patients were excluded due to loss to follow-up or non-adherence during the study period. As presented in Table 1, baseline characteristics were comparable between groups, except for a higher proportion of P-CAB use in the genotype-guided group (59.0% vs. 46.5%, *P* = 0.002). The mean age was 45.2 ± 13.6 years in the empirical group and 47.6 ± 13.9 years in the genotype-guided group (*P* = 0.074). Male patients accounted for 38.3% and 40.6%, respectively (*P* = 0.562). Smoking and alcohol consumption histories were similar between groups (both *P* > 0.05).

**TABLE 1:** Baseline characteristics.

Characteristic	Empirical group (n = 355)	Genotype-guided group (n = 261)	*P*
Age (years, mean ± SD)	45.2 ± 13.6	47.6 ± 13.9	0.074
Male, n (%)	136 (38.3)	106 (40.6)	0.562
Smoking, n (%)	70 (19.7)	54 (20.7)	0.765
Alcohol consumption, n (%)	92 (25.9)	77 (29.5)	0.323
P-CAB-based regimen, n (%)	165 (46.5)	154 (59.0)	0.002
BMI (kg/m^2^, mean ± SD)	24.1 ± 3.8	24.4 ± 4.4	0.421

Smoking defined as current daily or weekly consumption of ≥1 cigarette per day for ≥6 months; alcohol consumption defined as intake of ≥50 g of pure alcohol per week for ≥6 months. P-CAB, potassium-competitive acid blocker (keverprazan 20 mg or tegoprazan 50 mg).

### Eradication rates

As shown in Table 2, the overall eradication rate was significantly higher in the genotype-guided group compared with the empirical group (237/261 [90.8%] vs. 290/355 [81.7%], unadjusted *P* = 0.001). Multivariate analysis confirmed genotype-guided therapy as an independent predictor of eradication success (OR 2.03, 95% CI 1.23–3.36, adjusted *P* = 0.006), after adjusting for age, sex, smoking, alcohol consumption, and acid suppressant type. In the empirical group, P-CAB-based regimens achieved significantly higher eradication rates than PPI-based regimens (145/165 [87.9%] vs. 145/190 [76.3%]; OR 2.24, 95% CI 1.38–3.58; *P* < 0.001). In contrast, in the genotype-guided group, the difference between P-CAB-based and PPI-based regimens was not statistically significant (144/154 [93.5%] vs. 93/107 [86.9%]; OR 2.12, 95% CI 0.94–4.78; *P* = 0.070).

**TABLE 2:** Eradication rates and impact of acid suppressant type.

Analysis	Eradication rate	OR (95% CI)	*P*
Overall (genotype-guided vs. empirical therapy)			
Treatment strategy	237/261 (90.8%) vs. 290/355 (81.7%)	2.03 (1.23–3.36)	0.006^a^
Acid suppressant type (P-CAB vs. PPI)			
Empirical group	145/165 (87.9%) vs. 145/190 (76.3%)	2.24 (1.38–3.58)	<0.001
Genotype-guided group	144/154 (93.5%) vs. 93/107 (86.9%)	2.12 (0.94–4.78)	0.070

PPI: proton pump inhibitor (esomeprazole 20 mg or ilaprazole 5 mg). Eradication rates are presented as number of successes/total patients (percentage).

^a^ Adjusted *P*-value from multivariate logistic regression analysis controlling for age, sex, smoking, alcohol consumption, and acid suppressant type. The unadjusted *P*-value for the bivariate comparison was 0.001.

### Resistance patterns and eradication outcomes

Among the 261 patients receiving genotype-guided therapy, the resistance profiles included 101 patients (38.7%) with full susceptibility to both antibiotics, 66 (25.3%) with clarithromycin resistance only, 24 (9.2%) with levofloxacin resistance only, and 70 (26.8%) with dual resistance to both agents (Table 3). Dual resistance was associated with the lowest eradication rate (58/70 [82.9%]), significantly lower than that in fully susceptible patients (95/101 [94.1%]) after multivariate adjustment (OR 0.30, 95% CI 0.11–0.86, *P* = 0.025). In contrast, patients with single-drug resistance achieved favorable eradication (clarithromycin-resistant: 61/66 [92.4%]; levofloxacin-resistant: 23/24 [95.8%]). However, the confidence intervals for these estimates were wide (clarithromycin-resistant: OR 0.74, 95% CI 0.21–2.56; levofloxacin-resistant: OR 1.31, 95% CI 0.15–11.54), reflecting the limited sample sizes in these subgroups. These findings should therefore be interpreted with appropriate caution, and the apparent comparability to the fully susceptible group should not be overinterpreted.

**TABLE 3:** Eradication rates by resistance pattern in the genotype-guided group.

Resistance pattern	n (%)	Eradication (n/N, %)	OR (95% CI)	*P*
Fully susceptible	101 (38.7)	95/101 (94.1)	1.00 (References)	—
Clarithromycin-resistant	66 (25.3)	61/66 (92.4)	0.74 (0.21–2.56)	0.634
Levofloxacin-resistant	24 (9.2)	23/24 (95.8)	1.31 (0.15–11.54)	0.810
Dual-resistant	70 (26.8)	58/70 (82.9)	0.30 (0.11–0.86)	0.025

### Factors associated with eradication failure

As shown in Table 4, in subgroup analysis limited to the genotype-guided group, multivariate analysis revealed that alcohol consumption was independently associated with a lower likelihood of eradication (OR 0.39, 95% CI 0.16–0.92, *P* = 0.032). Dual resistance (compared with full susceptibility) remained a significant independent predictor of treatment failure (OR 0.30, 95% CI 0.11–0.86, *P* = 0.025). Acid suppressant type did not reach statistical significance in this subgroup (OR 2.12, 95% CI 0.94–4.78, *P* = 0.070).

**TABLE 4:** Multivariate analysis of factors associated with eradication failure in the genotype-guided group.

Variable	OR (95% CI)	*P*
Alcohol consumption		
Yes vs. no	0.39 (0.16–0.92)	0.032
Resistance pattern		
Dual-resistant vs. fully susceptible	0.30 (0.11–0.86)	0.025
Clarithromycin-resistant vs. fully susceptible	0.74 (0.21–2.56)	0.634
Levofloxacin-resistant vs. fully susceptible	1.31 (0.15–11.54)	0.810
Acid suppressant		
P-CAB vs. PPI	2.12 (0.94–4.78)	0.070

All multivariate models were adjusted for the full covariate set. Variables were selected based on univariate *P* < 0.10, with age and sex forced into the model as clinically relevant confounders. Interaction terms between treatment strategy and acid suppressant type, and between resistance pattern and acid suppressant type in the genotype-guided group, were tested and were not statistically significant (all *P* > 0.05).

## Discussion

This single-center retrospective cohort study demonstrated that genotype-guided therapy based on qPCR detection of clarithromycin and levofloxacin resistance significantly improved eradication rates compared with empirical therapy. The superiority of P-CAB over PPI was most pronounced in the empirical setting, whereas this advantage was not statistically significant in the genotype-guided group. Notably, dual resistance to clarithromycin and levofloxacin emerged as a powerful independent predictor of treatment failure even with genotype-guided therapy, reducing eradication rates to below 85%. In contrast, single-drug resistance did not compromise eradication when the corresponding antibiotic was avoided. Additionally, alcohol consumption was identified as an independent risk factor for treatment failure in the genotype-guided group. These findings provide clinically actionable insights for optimizing *H. pylori* eradication in an era of rising antimicrobial resistance.

The global decline in *H. pylori* eradication rates has been primarily driven by escalating antimicrobial resistance, particularly to clarithromycin and levofloxacin (Schulz et al., 2025). A recent meta-analysis (Li C. et al., 2025) reported that genotype resistance-guided tailored therapy achieved pooled eradication rates of 84.4% by intention-to-treat analysis and 91.7% by per-protocol analysis, with superior efficacy compared to empirical therapy. Our findings extend this evidence by demonstrating that genotype-guided selection within BQT yields an eradication rate of 90.8%, significantly higher than empirical BQT (81.7%). The eradication rate in the genotype-guided group approaches the desirable target of ≥90% recommended by the Maastricht VI/Florence consensus, whereas the empirical group falls short (Malfertheiner et al., 2022). Our findings are broadly consistent with the randomized trial evidence reported by Salehi et al. (2025), which reported eradication rates of 82.2% for omeprazole-amoxicillin-clarithromycin regimen, 91.3% for omeprazole-amoxicillin-metronidazole-bismuth regimen, and 97.3% for omeprazole-amoxicillin-levofloxacin regimen in treatment-naïve patients with dyspepsia or peptic ulcer disease. This observation aligns with our finding that clarithromycin resistance is a major driver of treatment failure and that genotype-guided avoidance of clarithromycin in resistant patients can restore high eradication rates.

P-CABs represent a significant advance in acid suppression, offering more potent, rapid, and sustained inhibition of gastric acid secretion independent of CYP2C19 genotype (Shang et al., 2025). Our finding that P-CAB was superior to PPI in empirical therapy (87.9% vs. 76.3%, *P* < 0.001) aligns with accumulating evidence (Agago et al., 2024; Ouyang et al., 2024). A meta-analysis (Zhang et al., 2025) comparing tegoprazan-based versus PPI-based regimens reported comparable eradication rates (78.6% vs. 76.6%, *P* = 0.31) but significantly lower adverse events with P-CAB. However, our study demonstrates that the magnitude of P-CAB benefit depends critically on the treatment context. In the genotype-guided group, where antibiotic selection was optimized, the P-CAB advantage was attenuated and no longer statistically significant. This observation suggests that when antibiotics are appropriately matched to susceptibility, the contribution of acid suppression may be less critical to achieving eradication (Borody et al., 2023). Nevertheless, the numerically higher eradication rate with P-CAB (93.5% vs. 86.9%) suggests a potential clinical benefit that may reach significance in larger studies.

There was a baseline imbalance in the use of P-CABs between the two groups, with a significantly higher proportion of P-CAB use in the genotype-guided group (59.0% vs. 46.5%). This imbalance could theoretically bias the results in favor of genotype-guided therapy, given the superior efficacy of P-CAB observed in the empirical setting. To address this concern, we included P-CAB type as a forced covariate in all multivariable models and performed stratified analyses by acid suppressant type, which demonstrated consistent genotype-guided effects across both PPI and P-CAB subgroups. Furthermore, the E-value analysis confirmed that the observed treatment effect is against unmeasured confounding: an unmeasured confounder would need to be associated with both treatment assignment and outcome by a risk ratio exceeding 2.81 to fully eliminate the observed association. Given the measured covariates, such a strong unmeasured confounder is highly implausible. Nevertheless, the observational nature of this study precludes definitive causal inference, and our findings warrant confirmation in randomized controlled trials.

The present study found that patients with clarithromycin-only resistance (92.4%) and levofloxacin-only resistance (95.8%) achieved eradication rates comparable to those of fully susceptible patients (94.1%). This demonstrates that genotype-guided therapy effectively neutralizes the impact of single-drug resistance, supporting the clinical utility of resistance testing even in settings where dual resistance is not universal. In contrast, dual resistance emerged as a powerful independent predictor of treatment failure, reducing eradication to 82.9%. The persistent failure in dual-resistant patients, even with genotype-guided therapy, underscores several points. First, current treatment options for dual-resistant *H. pylori* remain suboptimal. In our study, these patients received alternative regimens avoiding both clarithromycin and levofloxacin, including amoxicillin-furazolidone, tetracycline-metronidazole, or doxycycline-metronidazole combinations. However, eradication rates remained below 85%, suggesting that these regimens may have intrinsic limitations or that resistance to other antibiotics may be present but not tested. Second, the failure in dual-resistant patients highlights the need for expanded susceptibility testing beyond clarithromycin and levofloxacin. Third, these patients may benefit from alternative strategies such as high-dose dual therapy, which has shown promise in salvage settings (Hu et al., 2023).

Alcohol consumption was identified as an independent predictor of eradication failure in the genotype-guided group. This finding is consistent with previous studies identifying lifestyle factors as modifiable determinants of treatment outcome (Zhang et al., 2018; Shah et al., 2021). However, because medication adherence was not systematically captured in this retrospective study, the observed association between alcohol consumption and treatment failure may be partially or fully mediated by alcohol-related non-adherence. Alcohol consumption is well recognized as a predictor of poor medication adherence across multiple chronic disease contexts, and patients who consume alcohol may be less likely to adhere to complex multidrug regimens requiring multiple daily doses (Malfertheiner et al., 2022). Alcohol may also directly affect the gastric environment, potentially altering drug absorption or the activity of antibiotics (Lieber, 1998). Additionally, alcohol use is often associated with other lifestyle factors that may independently influence treatment outcomes (Ikezaki et al., 2017). The persistence of this effect even in the genotype-guided group, suggests that adherence and behavioral factors remain critical determinants of success, though the relative contribution of adherence versus direct biological mechanisms cannot be determined from the present data. Clinically, this finding emphasizes the importance of counseling patients about alcohol cessation during eradication therapy, particularly in patients receiving complex regimens, while also highlighting the need for prospective studies with systematic adherence monitoring to confirm the independent nature of this association. Future prospective studies should incorporate comprehensive assessments of lifestyle, behavioral, and socioeconomic factors to better understand the determinants of eradication success.

Several limitations merit consideration. First, the significant limitation is the retrospective, non-randomized design of this study. Treatment allocation was determined by routine clinical practice rather than random assignment, and although we adjusted for measured confounders in multivariable models and performed E-value calculations to assess sensitivity to unmeasured confounding, we cannot exclude the possibility of residual confounding by unmeasured or imprecisely measured factors. For instance, physicians may have preferentially offered genotype-guided testing to patients with specific clinical characteristics, or temporal changes in practice may have introduced systematic differences between the groups. While we enrolled patients consecutively and observed comparable baseline characteristics for most variables, the higher P-CAB use in the genotype-guided group suggests that practice patterns evolved over time, which may reflect a temporal confounding effect. The observational nature of this study precludes definitive causal inferences, and our findings should be interpreted as associations requiring confirmation in randomized controlled trials. Second, treatment adherence is one of the strongest determinants of *H. pylori* eradication success, and non-adherence is a frequent cause of eradication failure (Zeng et al., 2023). Differential adherence between the genotype-guided and empirical therapy arms may have biased the observed treatment effect. Additional testing and counseling in the genotype-guided group could have enhanced adherence through a Hawthorne effect (Sedgwick and Greenwood, 2015). Without objective adherence measures, we cannot quantify its contribution to the between-group difference in eradication rates. Although E-value analysis indicates that eliminating the observed association would require strong unmeasured confounding, an effect of adherence disparities cannot be fully excluded. This limitation has also implications for the interpretation of the finding for alcohol consumption, as we were unable to determine whether the observed association represents a direct biological effect of alcohol, a consequence of alcohol-related non-adherence, or residual confounding by other lifestyle factors. Additionally, other potential confounding variables, including CYP2C19 genotype, dietary factors, socioeconomic status, diabetes mellitus, prior peptic ulcer disease, prior antibiotic exposure, concomitant medications, and detailed comorbidities, were not adjusted for and may have biased the observed associations. Third, this study employed a per-protocol analysis; an intention-to-treat analysis was not performed due to the retrospective design and exclusion of patients with protocol deviations. Fourth, qPCR assay detected resistance only to clarithromycin and levofloxacin; resistance to other antibiotics was not assessed. Fifth, the sample size for certain subgroup analyses, particularly levofloxacin-resistant only patients and specific dual-resistant treatment regimens, was limited, reducing statistical power for these comparisons. Consequently, the apparent comparability of single-drug resistance eradication rates to those of fully susceptible patients should be interpreted with caution. Future studies with larger sample sizes are needed to provide more precise estimates for these subgroups. Sixth, while our findings demonstrate clinical superiority of genotype-guided therapy, we did not evaluate health-economic outcomes, including cost-effectiveness analysis, testing turnaround time, or the accessibility of qPCR testing in different healthcare settings. Patient-centered outcomes such as treatment satisfaction, quality of life, or willingness to undergo testing were also not assessed. Future prospective studies incorporating comprehensive health-economic and patient-reported outcomes are needed to determine the optimal integration of genotype-guided therapy into clinical practice.

## Conclusion

In this retrospective cohort study, genotype-guided therapy based on qPCR detection of clarithromycin and levofloxacin resistance significantly improved *H. pylori* eradication rates compared with empirical bismuth-containing quadruple therapy. The benefit of P-CAB over PPI was most pronounced in the empirical setting. Dual resistance to clarithromycin and levofloxacin emerged as a powerful independent predictor of treatment failure even with genotype-guided therapy, whereas single-drug resistance showed favorable eradication when the corresponding antibiotic was avoided. However, these findings should be interpreted with appropriate caution, and larger studies are needed to provide more precise estimates. These findings support the clinical consideration of genotype-guided therapy in settings where qPCR testing is available. However, health-economic evaluations and assessments of patient-centered outcomes are needed before recommending widespread adoption.

## Data Availability

The original contributions presented in the study are included in the article/supplementary material, further inquiries can be directed to the corresponding author.
